# 

**Published:** 1856-12

**Authors:** 


### INDEX TO VOL. V.

PAGE.
Adams Wm. on Infantile Paralysis,............................................................ 72
Address, Valedictory, by T. D. Washburn,..................................................103
Address, Valedictory, by W. B. Herrick,......................................................199
Address of the President of American Medical Association, 1856,..............379
Address on Alcoholic Drinks in Phthisis, by N. S. Davis,........................... 406
Albumenuria, or Bright's Disease,.........................................487, 130, 42
Alkaline Method of Treatment in Acute Rheumatism,..................................455
Alsidium Blodgettii in Consumption and Scrofula,......................................483
American Medical Association, History of,....................................................45
American Medical Association, Annual Meeting of,.............475, 259, 193, 100
Amerman, George, on Laryngotomy,...........................................................439
Amputation of Penis,....................................................................................422
Amputation of the Thigh, by H. A. Johnson,................................................442
Apology, ........................................................................................................53
Æsculapian Society, Proceedings of,.................................568, 327, 193, 21

Barlow, George Hilaro, on Practice of Medicine,..........................................312
Beckner, J. F. Cases of Puerperal Convulsions,..............................................62
Bennett on Uterine Pathology,.......................................................................524
Blakiston, Peyton, on the Treatment of Sciatica,.............................................75
Brainard, D. Operation for Inguinal Hernia,.....................................................94
Bronchitis and Pleurisy, with Effusion, Pneumothorax, &c............................452
Bronzed Skin from Disease of Supra-Renal Capsules,....................................243
Bromine as a Specific in Pseudo-Membranous Affections,.............................485
Brown, Isaac Baker, on Surgical Diseases of Woman,....................................313
Byford, W. H. on Milk-Sickness or Tires,.........................................................25

Cancer of Penis, Amputation of,......................................................................422
Carbonic Acid Gas as a Local Anæsthetic in Uterine Diseases,......................425
Cases in Practice, by Benj. Woodward,............................................................59
Chemistry, Physiological, by C. G. Lehmann,...................................................41
Chloroform, Remote Effects of,.......................................................................292
Cholera Epidemic, Treatise on, by H. G. Jameson, Sen.....................................84
Cholera in Moscow, Portugal, and Madeira...................................482, 485
Chronic Dysentery, Benzoin in Treatment of,.................................................533
Churchill, Fleetwood, on the Diseases of Infants and Children,.....................566
Cinchonæ, Sulphate of, in Intermittents,.......................................................514
Climate of Lake Superior, by Charles Whittlesey,.............................................55
Clinic of Dr. Biddle,.........................................................................................130
Clinical Lectures on Surgery, by M. Nelaton,.....................................................38
College of Physicians and Surgeons of New York,............................................54
Commencement in Rush Medical College,.........................................................98
Consumptive Hospital in New York,...................................................................54
Consumption, Sun's Rays in,.............................................................................532
Controversy, .....................................................................................................336
Cook County Medical Society, Proceedings of,..............................195, 87, 42
Cornea, Laceration of,......................................................................................305
Curling, F. B. on the Testes, &c.......................................................................474

Davis, N. S. on the Means of Preserving Milk, &c...............................................7
Davis, N. S. on History of American Medical Association,..................................45
Davis, N. S. on Alcoholic Drinks in Phthisis,....................................................406
Davis, N. S. on Changes in the Composition and Properties of the Milk of the Human Female, Produced by Menstruation and Pregnancy,.........535
Davis, H. W. on Dysentery,...............................................................................295

Death from Drinking Naphtha,..........532
Delaware County Medical Society,..........241
DeWitt County Medical Society,..........146
Dictionary, Medical, by R. D. Hoblyn,..........40
Disease, Prevalence of,..........145
Dislocations, Sprains, and Fractures, Effects of,..........161
Dissector's Manual, &c. by Erasmus Wilson,..........525
Doctor's Life, by an M.D..........102
Dropsy, Abdominal,..........127
Dysenteria Intermittent,..........295
Dysentery,..........479

Editors, Association of,..........197
Empyema, Case of,..........91
Erysipelas, Treatment of by Proto-Sulphate of Iron,..........101
Essay for a Prize,..........529
Excrescences, Warty, of the Labia,..........89
Experimental Physiology, by M. Bernard,..........218
Extra-Uterine Pregnancy,..........376
Extensive Injury During Pregnancy,..........531

Fever, Yellow, by Theod. Walzer,..........151
Flint, Austin, on Physical Exploration and Diagnosis,..........463
Fœtal Malformations, by W. W. Adams,..........215
Forceps *versus* Embryulcia,..........291
Fox, G. M. on Injury to Perinium,..........115
Fracture, Compound, of the Thigh, by D. Prince,..........125
Fractures, Ununited,..........458
Freer, J. W. on Fractures, &c..........161
Freese, J. R. on Forceps *versus* Embryulcia,..........450, 252

Gangrene from Cold,..........101
Graduates of Rush Medical College,..........99
Graduates of Medical Colleges,..........246
Graham, James, on Alkalies in Rheumatism,..........455
Goodbrake, C. on Pre-Expulsion of Placenta,..........211
Goodwin, A. E. on Intermittents and Their Remedies,..........18
Glycerine, an Inaugural Thesis, by H. Wardner,..........247

Hall, W. G. on Neuro-Pathology of Fever,..........213
Heart, Movements of, Indicated by a New Instrument,..........484
Heart and Aorta, Diseases of, by Wm. Stokes,..........86
Heise, A. W. on Extra-Uterine Pregnancy,..........376
Henry County Medical Society,..........334, 195
Hernia, Inguinal, with Operation by D. Brainard,..........94
Herrick, W. B. Valedictory Address,..........199
Hoblyn, Richard D., Dictionary of Terms, &c..........40
Human Physiology, by Robley Dunglison,..........475

Illinois State Medical Society, Transactions of,..........318, 181, 53
Infants and Children, Diseases of, by Fleetwood Churchill,..........566
Inhalation of Medicated Vapors,..........87
Injuries of Shoulder Joint, Diagnosis of,..........519
Insanity, &c. in France,..........54
Intermittent Insanity, Case of..........84
Introduction to Practical Pharmacy, by Edward Parrish,..........36
Iodide of Potassium in the Treatment of Ague,..........428
Isham, Ralph, Case of Bronchitis, Pleurisy, &c..........452
Itch, Cure of, in Half an Hour,..........482

Jameson, H. G. Sen. on Epidemic Cholera,........84

Johnson, H. A. on Albumenuria,........42

Knee-Joint, Injury of—Amputation, &c........442
Kreider, H. W. on Quinine for Intermittent Insanity,........64

Labor Complicated, by Hiram Nance,........493
Lake Superior, Climate of,........55
Laryngotomy After Apparent Death, by G. Amerman,........439
Legal Responsibilities,........531
Lehmann, C. G. Physiological Chemistry,........41
Letters to a Young Physician, by James Jackson,........81
Life, Duration of,........80
Long, S. O. on Paralysis, ........256
Louisville Review,........337

Manson, Wm. and Richardson, John T. on Case of Compound Fracture of the Skull, with Symptons of Compression—Operation,........547
McKinney, J. W. on Laceration of the Cornea,........305
Medical Education—Lengthened College Terms, &c........138
Medical Profession of Ancient Times, by John Watson,........433
Medical Knowledge, by J. R. Freese,........450
Medical Journals, Number of,........573
Medical Ethics,........574
Medical Jurisprudence, by A. Taylor,........525
Medicines, Patent,........337
Meningitis, Treatment of, by J. W. McKinney,........118
Milk, Modes of Preservation,........7
Milk of the Human Female, Report on the Changes in Composition and Properties, Produced by Menstruation and Pregnancy, by N. S. Davis, 535
Milk Sickness,........25
Miller, James, on the Principles of Surgery,........312
Mills, J. R. on the Use of Veratrum Viride,........16
Morfit, J. C. on Poisoning by Strychnine,........443
Mortality in the French Army During the War in the East,........480
Mortality, Statistics of,........429, 47

Nelaton, M. Clinical Surgery,........38
Neuro-Pathology, by G. W. Hall,........213
Norcum, F. B. on Pleuro-Tuberculosis,........307
Norcum, F. B. on Albumenuria,........487

Obstetric Memoirs, &c. by J. Y. Simpson, M.D........41
Obstetric Memoirs and Contributions of James Y. Simpson,........564
Occiput, Singular Effects of a Blow Upon,........448
Occupation, Influence of, on Mortality,........429
Opthalmic Medicine and Surgery, by T. Wharton Jones,........313

Paralysis, Cases of,........256
Paralysis, by A. W. Dewey,........239
Parrish, Edward, on Practical Pharmacy,........36
Perinium, Injury of, Puncture of the Bladder, &c........115
Personal Matters,........238
Pharmaceutical Association, Proceedings of,........135
Physical Exploration and Diagnosis, by A. Flint,........468
Placenta, Pre-Expulsion of,........211
Pleuro-Tuberculosis, by F. B. Norcum,........307
Pregnancy, Extra-Uterine,........290
Presidency of American Medical Association,........475

Professional Changes,....................................................................336
Professional Reputation,..................................................................533
Prince, David, Case of Compound Fracture of the Thigh,........................125
Puerperal Convulsions, Cases of,..........................................................62
Pustular Eruption of the Skin, from the Internal Use of Tartar Emetic,......101

Ranking's Half Yearly Abstract,..........................................................134
Regurgitation, Obstinate, Treated by Chloroform, by Isaac E. Taylor,......551
Report on Meteorological Characteristics, &c. for 1856,..........................497
Report on Practical Medicine, by Samuel Thompson,.........................391,343
Rush Medical College, Public Commencement of,......................................98
Rush Medical College,...........................................................573, 479, 480

Scarlatina, Treatment of by Oil of Turpentine,.......................................148
Sciatica, Treatment of, by Peyton Blakiston,...........................................75
Sherman, N. on Cancer of Penis,..........................................................422
Simpson, James Y. Obstetric Memoirs and Contributions of,....................564
Skull, Compound Fracture of, with Symptoms of Compression—Operation,
by Wm. Manson and John T. Richardson,...........................................547
Statistics of Mortality,..........................................................................47
Sterility, by A. K. Gardner,....................................................................524
Subscribers, Notice to,.......................................................574, 480, 1 9
Surgery, Principles of, by James Miller,..................................................312
Surgical Practice, Treatise on, by H. H. Smith,........................................525
Surgical Diseases of Women, by I. B. Brown,..........................................313
Sydenham Society, Works Published,....................................................534
Syphilis by Vaccine Lymph,....................................................................18

Taylor, Isaac E. Case of Obstinate Regurgitation Treated by Chloroform, 551
Tetanus, ...........................................................................................509
Therapeutics and Pharmacology, by George B. Wood,..............................567
Thompson, Samuel, on Practical Medicine,...................................391, 343
Thompson, Allan, on Origin of Entozoa,.................................................521

Union Medical Association,..................................................................436
Unprofessional Advertisements,...........................................................435
Ununited Fractures,............................................................................530
Uterine Hemorrhage, Treatment of,.........................................................69

Vaccination in Relation to Blindness,.....................................................484
Veratrum Viride in Fevers,....................................................................16
Very Sensitive,...................................................................................433

Wardner, H. on Glycerine,...................................................................247
Washburn, T. D. Valedictory Address, &c...............................................103
Watson, John, on the Medical Profession of Ancient Times,.......................433
Willard, E. R. on Turning with Rigid Os-Uteri,.........................................423
Wood, George B. on Therapeutics and Pharmacology,...............................567
Woodward, Benjamin, on Cases in Practice,...............................................59

"Germans 58, Dutch 8, Swedish 18, English 30, French 34, Belgian 13, Italian 12, Spanish 9, Canadian 3, United States 40—total, 225. The Journals of Pharmacy are included in this list, of which there is a goodly number on both sides of the Atlantic; but it does not embrace the numerous journals, a large majority of which are published in this country, devoted to some one or another of the exclusive systems of practice in vogue."

---

*To Subscribers.*

The present number of the *Journal* closes the fifth volume of the new series, and there are still many subscribers who have not paid their subscriptions. Indeed, there is over $1,500 due us from subscribers to Volumes IV. and V. Will not each individual concerned give immediate attention to this matter. We would like to commence the New Year with all previous accounts settled, and the books fairly balanced.

---

*Medical Ethics.*

Rush Medical College, ........ 573

Number of Medical Journals, ........ 573

To Subscribers, ........ 574

Medical Ethics, ........ 574

### RECEIPTS ON SUBSCRIPTIONS,

UP TO DEC. 1ST, 1856.

### ILLINOIS.

| | | |
|---|---|---|
| Dr. Mergler, . | Vol. 5, | $1,00 |
| " T. O. Washburn, | " 5, | 2,00 |
| " W. F. Vermilion, | " 5, | 2,00 |
| " F. S. Miner, | Vol. 4 & 5, | 4,00 |
| " D. Prince, . | Vol. 5, | 2,00 |
| " E. Friend, . . | " 5, | 2,00 |
| " B. S. Lewis, | Vol. 4 & 5, | 4,00 |

### MICHIGAN.

| | | |
|---|---|---|
| Dr. R. S. Hawley, | Vol. 5, | $2 00 |
| " Wm. Upjohn, | Vol. 4 & 5, | 4,00 |

### IOWA.

| | | |
|---|---|---|
| Dr. J. N. B. Elliott, | Vol. 5, | $2,00 |

### WISCONSIN.

| | | |
|---|---|---|
| Dr. G. W. Lee, | Vol. 4 & 5, | $4,00 |

### MINNESOTA TERRITORY.

| | | |
|---|---|---|
| Dr. J. H. Murphy, | Vol. 5, | $2,00 |
| " J. M. Winn, . | " 5, | 2,00 |

